# Hypoxia-Driven Extracellular Vesicles Promote Pro-Metastatic Signalling in LNCaP Cells via Wnt and EMT Pathways

**DOI:** 10.3390/biology14091135

**Published:** 2025-08-27

**Authors:** Melissa Santos, Khansa Bukhari, Irem Peker-Eyüboğlu, Igor Kraev, Dafydd Alwyn Dart, Sigrun Lange, Pinar Uysal-Onganer

**Affiliations:** 1 Cancer Mechanisms and Biomarkers Research Group, School of Life Sciences, University of Westminster, London W1W 6UW, UK; m.santos@westminster.ac.uk (M.S.); w1977691@westminster.ac.uk (K.B.); pekerirem@gmail.com (I.P.-E.); 2 Department of Medical Biology, School of Medicine, Marmara University, Istanbul 34899, Turkey; 3 Electron Microscopy Suite, Faculty of Science, Technology, Engineering and Mathematics, Open University, Milton Keynes MK7 6AA, UK; igor.kraev@open.ac.uk; 4 UCL Cancer Institute, University College London, Paul O’Gorman Building, 72 Huntley Street, London WC1E 6DD, UK; a.dart@ucl.ac.uk; 5 Pathobiology and Extracellular Vesicles Research Group, School of Life Sciences, University of Westminster, London W1W 6UW, UK; s.lange@westminster.ac.uk

**Keywords:** prostate cancer, hypoxia, extracellular vesicles (EVs), tumour microenvironment, Wnt signalling (Wnt), epithelial–mesenchymal transition (EMT), invasion, HIF-1α

## Abstract

Prostate cancer (PCa) progression is shaped by the tumour microenvironment, where hypoxia promotes aggressiveness and contributes to therapy resistance. Extracellular vesicles (EVs), secreted under hypoxia, can deliver modified bioactive cargo that reprograms recipient cells. This study examined whether EVs from hypoxia-conditioned metastatic PCa cells enhance malignant traits in cancerous and non-tumorigenic prostate cell lines via Wnt signalling and epithelial–mesenchymal transition (EMT). EVs from PC3 cells cultured under hypoxia (1% O_2_) or normoxia (21% O_2_) as control were applied to LNCaP (low metastatic potential) and PNT2 (non-tumorigenic) cells. PC3 hypoxia-derived EVs increased HIF-1α, upregulated mesenchymal markers (*Vimentin*, *N-cadherin*) and Wnt-related genes (*Wnt3A*, *Wnt5A*, *Fzd7*), and suppressed the epithelial marker *E-cadherin*. Functional assessment showed that LNCaP cells treated with PC3 hypoxia EVs showed greater motility and invasiveness, and PNT2 cells displayed transcriptomic reprogramming. These findings show that hypoxia-driven EVs can propagate pro-metastatic signalling in less aggressive and normal prostate cells. The findings highlight EVs as a potential therapeutic target in PCa progression.

## Simple Summary

Prostate cancer is one of the most common cancers in men and can become life-threatening when it spreads to other parts of the body. Reduced oxygen levels, or “hypoxia,” are often found inside tumours and are known to change cancer cells’ characteristics, making them more aggressive and harder to treat. In this study, extracellular vesicles (EVs), which are small lipid particles released by cells and carry messages that can affect how other cells behave, were collected from prostate cancer cells that had been grown in hypoxic conditions. When these EVs were applied to less aggressive prostate cancer cells, the recipient cells began to behave more like the aggressive ones, showing changes in gene pathways linked to cell movement and cancer spread. This suggests that hypoxic cancer cells can send signals via EVs that make surrounding cells more dangerous. Disrupting this EV-mediated communication could help slow or prevent prostate cancer progression and improve treatment outcomes.

## 1. Introduction

Cancer is one of the leading causes of morbidity and mortality worldwide, and its incidence continues to rise despite advances in detection and treatment. Early identification and understanding of cancer biology are critical for improving patient outcomes. Among malignancies affecting men, prostate cancer (PCa) is of particular concern, ranking as the second most frequently diagnosed cancer and a major cause of cancer-related death globally [1,2]. Several methods are currently used to detect and diagnose PCa, each with distinct advantages and limitations. While prostate-specific antigen (PSA) testing remains the most widely applied screening tool, its limited specificity can lead to overdiagnosis and unnecessary biopsies. Digital rectal examination (DRE) offers a low-cost clinical assessment but has poor sensitivity, particularly for early-stage disease. Multiparametric magnetic resonance imaging (mpMRI) has improved lesion localisation and risk stratification; however, its interpretation requires specialist expertise and access to advanced imaging facilities. Prostate biopsy, the diagnostic gold standard, is invasive and subject to sampling error, which can lead to under- or overestimation of tumour grade and stage. These limitations highlight the need for additional, reliable biomarkers and novel diagnostic approaches to improve detection accuracy and guide clinical decision-making [3]. Metastatic progression, particularly to bone, significantly worsens prognosis and correlates with therapy resistance and poor outcomes [2,4]. One of the critical microenvironmental factors that drives this malignancy is hypoxia, defined as a condition of reduced oxygen availability in tissues [5]. Hypoxia is a hallmark of solid tumours, resulting from rapid tumour growth that outpaces the development of an adequate blood supply. This leads to spatial and temporal heterogeneity in oxygen distribution within the tumour microenvironment (TME), promoting tumour progression, therapy resistance, and metastatic dissemination [6].

The primary cellular response to hypoxia is orchestrated by the hypoxia-inducible factors (HIFs), particularly *HIF-1α*, which serve as master regulators of the transcriptional adaptation to low oxygen conditions [7]. Upon activation, HIFs regulate a wide range of genes involved in angiogenesis, metabolic reprogramming, invasion, and epithelial–mesenchymal transition (EMT), all of which contribute to a more aggressive cancer phenotypes [8]. Notably, in PCa, hypoxia has been shown to upregulate EMT-inducing transcription factors promoting cadherin switching and the expression of mesenchymal markers, including *Vimentin* (*Vim*), thereby enhancing cellular motility and invasion [9].

Wnt signalling plays a central role in development, stem cell maintenance, and cancer progression [10,11]. Among the Wnt-related genes relevant to PCa, *Wnt3a*, *Wnt5a*, *Fzd7*, *sFRP1*, and *ROR2* stand out due to their roles in modulating tumour behaviour under hypoxic conditions [12,13,14]. *Wnt3a* is associated with β-catenin-dependent signalling and is implicated in promoting proliferation and metastasis, with its expression potentially regulated by HIFs [7]. Conversely, *Wnt5a* signals through non-canonical pathways and has been reported to exert both pro- and anti-tumour effects depending on cellular context; in PCa, it promotes migration and invasion via kinases such as Jnk [15]. *Fzd7*, a receptor for Wnt ligands, is upregulated in various malignancies, including PCa, where it plays a pivotal role in tumour progression [16]. In PCa, elevated *Fzd7* expression has been associated with the activation of both canonical and non-canonical Wnt signalling pathways, contributing to increased cellular plasticity, EMT, and enhanced motility. Importantly, *Fzd7* has also been associated in resistance to androgen deprivation therapy by supporting the survival of PCa stem-like cells and promoting lineage plasticity [17]. These properties not only facilitate tumour persistence under therapeutic challenges but also contribute to metastasis and recurrence. Therefore, *Fzd7* could be a potential biomarker of aggressive disease and a candidate for targeted therapies for PCa [18,19].

*sFRP1*, a Wnt pathway antagonist, is often silenced in PCa through epigenetic mechanisms, and its repression contributes to Wnt-driven oncogenicity [20,21]. *ROR2*, also a non-canonical Wnt receptor, has been implicated in tumour invasiveness, although its direct link with hypoxia in PCa still requires further studies [22].

EVs are released from cells as a critical contributor in cellular communication through the transport of their cargoes (including non-coding RNAs, mRNAs and proteins) and play key roles in tumour progression [23]. In PCa, EVs have been shown to facilitate key oncogenic processes such as immune evasion, therapy resistance, and establishment of the pre-metastatic niche [24]. Tumour-derived EVs can promote proliferation, migration, and EMT [23,24,25]. Notably, EVs derived from hypoxic PCa cells have been implicated in enhancing the metastatic potential of otherwise non-aggressive cells, suggesting a critical role in disease progression and therapeutic response [23,26]. Hypoxic conditions furthermore increase EV release and alter EV cargoes, including via HIF-mediated mechanisms [22]. In PCa, tumour-derived EVs have also been shown to enhance phenotypic plasticity and modulate immune interactions [24,26,27,28,29,30]. For example, EVs enriched with specific miRNAs such as miR-21 and miR-210, which are upregulated under hypoxia, were shown to enhance invasiveness and survival of PCa cells, indicating their central role in hypoxia-mediated tumour progression [29]. Therefore, a better understanding of the interplay between hypoxia, EV modulation, and EMT in PCa is essential for the development of novel targeted interventions. While ADT currently remains the standard treatment for advanced PCa, most patients eventually develop castration-resistant prostate cancer (CRPC), a form of disease that is associated with poor prognosis and limited therapeutic options [31,32]. Interestingly, resistance to ADT has been strongly associated with hypoxia within the TME [5,33,34]. Furthermore, PCa exhibits a predilection for metastasising to bone, where it encounters a unique microenvironment characterised by supportive stromal interactions and hypoxic niches [35,36]. The bone marrow niche not only facilitates tumour cell colonisation but also promotes survival and resistance through paracrine signalling and hypoxia-driven alterations in gene expression [37]. EVs are promising targets for therapeutic and diagnostic applications [23,27] and are being explored in early-phase clinical trials as delivery systems for precision therapeutics and immunomodulatory agents, due to their inherent stability, biocompatibility, and ability to target specific tissues [38]. Strategies aimed at disrupting EV biogenesis, modulating EV cargoes, or inhibiting EV uptake in recipient cells are also being investigated as potential therapeutic approaches to combat hypoxia-driven resistance and tumour progression in advanced PCa [39,40].

While previous studies have reported that EVs from PCa cells can influence tumour behaviour [41,42], roles for hypoxia-conditioned EVs in modulating Wnt signalling and EMT across both malignant and non-malignant prostate epithelial cells remain poorly understood. Most existing work has focused on single cell types or on normoxic conditions [43,44], leaving a critical gap in understanding how the hypoxic TME drives cellular communication promoting disease progression. The current study addresses this gap by assessing the molecular and functional effects of hypoxia-derived EVs from metastatic PC3 cells on both weakly metastatic (LNCaP) and non-tumorigenic epithelial cells (PNT2). Our data suggest a potential paracrine mechanism in which hypoxic tumour regions could influence surrounding cells toward a more invasive phenotype via EV signalling, which may contribute to tumour heterogeneity, therapy resistance, and metastasis [45,46]. These data can support further studies on EV-targeted strategies as a combinatory approach in PCa treatment.

## 2. Materials and Methods

### 2.1. Cell Culture

Three human prostate cell lines were used: non-tumorigenic epithelial PNT-2 (ATCC^®^ HPrEC, PCS-440-010) [47], weakly metastatic PCa LNCaP (ATCC^®^ CRL-1740) [48], and metastatic PC3 cells (ATCC^®^ CRL-1435) [49]. All cell lines were obtained from the American Type Culture Collection (ATCC, Manassas, VA, USA) and maintained in RPMI-1640 or Dulbecco’s Modified Eagle Medium (DMEM) supplemented with 10% foetal bovine serum (FBS) and 1% penicillin-streptomycin. Cells were cultured at 37 °C in a humidified incubator with 5% CO_2_ [50]. For hypoxic treatment, PC3 cells were incubated in a hypoxic chamber (1% O_2_, 5% CO_2_, 94% N_2_) for 6 h. Normoxic controls were maintained at ambient oxygen levels (21% O_2_).

### 2.2. EV Isolation and Characterisation

The isolation of EVs from PC3 cell cultures was performed using differential centrifugation and ultracentrifugation according to previously published studies [51] and adhering to the MISEV guidelines [52].

In brief, PC3 cells were cultured to 80% confluence in T75 flasks, the medium removed, the cells were washed with DPBS and fresh medium, without FBS (to avoid contamination with FBS-derived EVs), was applied for 6 h, and the flasks were kept at normoxia or hypoxia, respectively. Thereafter, the EV-containing media were collected and centrifuged at 4000× *g* for 20 min at 4 °C to remove cellular debris and aggregates. The EV-containing supernatants were then centrifuged at 100,000× *g* by ultracentrifugation for 1 h at 4 °C. The EV-enriched pellets were resuspended in 1 mL DPBS (filtered through a sterile 0.22 μM filter) and ultra-centrifuged again at 100,000× *g* for 1 h at 4 °C, after which the DPBS supernatant wash was discarded. The final EV pellets were resuspended in 50 μL DPBS and subjected to nanoparticle tracking analysis (NTA) for quantification and size profiling, to Western blotting for surface marker detection and imaged by transmission electron microscopy (TEM).

EVs were diluted 1:1000 in DPBS for NTA, concentration and size distribution were assessed using the NS300 NanoSight (Malvern Panalytical Ltd., Malvern, UK), equipped with a sCMOS camera and a 488  nm diode laser, with syringe speed set at 50, camera level for capture at 10 and for post-processing, the detection threshold was set at 5. Four 60 s videos were collected per sample, averaged and replicated in histograms (representing mean and standard error) using the NTA software (Malvern Panalytical Ltd., UK).

The EVs were assessed for two surface markers by semi-dry Western blotting using anti-CD63 (ab216130, Abcam, Cambridge, UK) and anti-flotillin-1 (ab41927), both diluted 1/1000 in TBS-T, after blocking in 5% BSA in TBS-T for 1 h at RT, with primary antibody incubation carried out overnight at 4 °C. Visualisation was carried out following secondary antibody incubation (HRP-labelled anti-rabbit IgG, BioRad, diluted 1/3000 in TBS-T) for 1 h at RT, using the UVP BioDoc-ITTM System (Thermo Fisher Scientific, Dartford, UK) with enhanced chemiluminescence (ECL, Amersham Biosciences, Buckinghamshire, UK).

For TEM, EVs were prepared as previously described [51] in 100 mM sodium cacodylate buffer (pH 7.4), applied to a glow-discharged TEM grid with a carbon support film, fixed with 2.5% glutaraldehyde (Agar Scientific Ltd, Stansted, UK) in 100 mM sodium cacodylate buffer, pH 7.4), and stained with 2% aqueous Uranyl Acetate (Agar Scientific Ltd, Stansted, UK). Digital images were captured using a GATAN Rio16 digital camera (Ametek GB Limited, Leicester, UK).

### 2.3. RNA Extraction and qRT-PCR for Hypoxia, Wnt Signalling and EMT-Related Gene Expression Changes

PNT2 and LNCaP cells were treated for 48 h (1.5 × 10^10^ particles/mL) with EVs collected from PC3 cells, which had been kept at normoxic (control) and hypoxic conditions, respectively. Following 48 h incubation of both cell lines with PC3-EV treatment, the downstream effects of the treatment on the cells were assessed.

RNA extraction from PC3 cell-derived EVs, LNCaP and PNT2 cells was performed using Trizol (Sigma, Haverhill, UK), with concentration and purity assessed by NanoDrop spectrophotometry (ThermoFisher Scientific, Hemel Hempstead, UK) at 260 nm and 280 nm absorbance. RNA was reverse transcribed into cDNA utilising the QuantiTect reverse transcription kit (Qiagen, Manchester, UK), with the resulting cDNA acting as the template for assessing the expression of the housekeeping gene *RPII*, and the target genes for analysis [53]. Primer sequences are presented in Table 1. The primers for *Ecad* and *Wnt3a* were designed and purchased from Sigma (Paisley, UK), while *RPII*, *Wnt5a*, *Fzd7* and *Vim* were obtained from Integrated DNA Technologies (IDT; Leuven, Belgium), and *HIF1α*, *sFRP1*, *ROR2* and *Vim* from ThermoFisher Scientific (Hemel Hempstead, UK).

### 2.4. Wound Healing and Invasion Assays

LNCaP cells were seeded onto a 12-well plate until a confluent monolayer was formed. Cells were treated with EVs (9.5 × 10^9^ EVs/mL) derived from the hypoxic and normoxic PC3 cells. LNCaP cells receiving no EV treatment were used as an additional control. A wound was performed on the cell monolayer using a p200 tip. The EVOS-FL auto (ThermoFisher Scientific, Hemel Hempstead, UK) system was used to photograph and measure the wound closure over a 48 h period under the environmental conditions of 5% CO_2_ at 37 °C. Each condition was performed in triplicate wells, and within each well, three points of reference were taken for the scratch. Semi-quantification of the wound area was measured using Image J software, version 1.51 (National Institutes of Health, Bethesda, MD, USA). Wound closure was calculated using the following equation: Motility index, MoI (%) = [1 − (wound width at given time/initial wound width)] × 100 as described before [57].

For invasion assays, 5 × 10^5^ LNCaP cells were seeded onto 8.0 µm pore diameter Corning Matrigel invasion inserts (Fisher Scientific, Loughborough, UK) in 24-well plates and treated with EVs as described above. Following overnight incubation, the percentage of invaded cells was assessed by staining with crystal violet (ThermoFisher Scientific, Hemel Hempstead, UK), and images were taken by EVOS-FL auto (ThermoFisher Scientific, Hemel Hempstead, UK) and quantified using ImageJ 1.54g (NIH, Bethesda, MD, USA) as previously described [51,58]. In parallel, the same number of cells was plated and incubated overnight to determine cell proliferation using an MTT (3-(4,5-dimethylthiazol-2-yl)-2,5-diphenyl tetrazolium bromide) assay, Absorbance was measured using a CLARIOstar plate reader (BMG Labtech, Aylesbury, UK) at 540–590 nm and normalised according to the non-treated/control cells. The experiments were performed three times from different biological samples with 3 technical repeats.

### 2.5. Statistical Analysis

GraphPad Prism v8.4 (La Jolla, CA, USA) was utilised for the statistical analysis. All experiments were performed in triplicate. The expression levels of genes were assessed by using Šídák’s test, ANOVA and Bonferroni multiple comparisons tests followed by Tukey’s post hoc analysis. Statistical significance was considered at *p* ≤ 0.05, with all results presented as mean ± SD.

## 3. Results

EVs isolated from the metastatic PC3 cells, incubated under normoxic versus hypoxic conditions, were used to assess their effects on both non-tumorigenic PNT2 and weakly metastatic LNCaP PCa cells. Changes in gene expression associated with hypoxia, Wnt signalling, and EMT, as well as alterations in cell motility and invasive potential, were assessed.

### 3.1. Hypoxia Enhances EV Secretion and Alters EV Profiles

**Figure 1:**
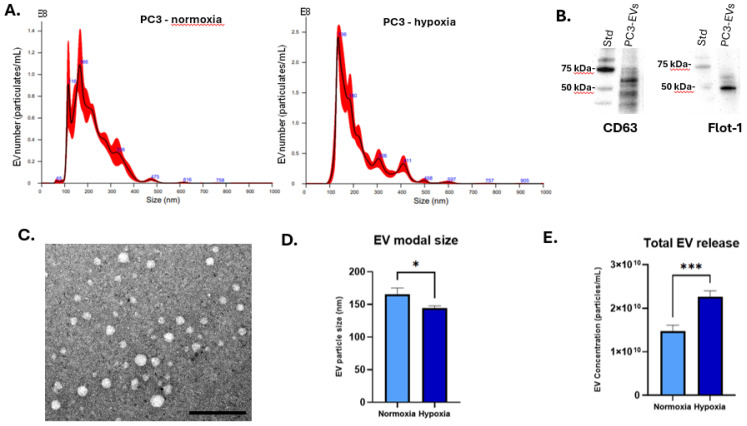
PC3 EV characterisation. (**A**) Nanoparticle tracking analysis (NTA) of EVs isolated from PC3 cells under normoxic or hypoxic conditions (6 h), showing size distribution profiles. (**B**) PC3-EVs are positive for CD63 and flotillin-1 as detected by Western blotting; size standard is indicated in kDa. (**C**) PC3 EVs imaged by TEM; the scale bar is indicated at 100 nm. (**D**) Modal size of PC EVs comparing normoxia and hypoxia-treated cells (6 h). (**E**) Total EV concentration is significantly increased in PC3 cells under hypoxic conditions (6 h). * *p* < 0.05; *** *p* < 0.001.

EVs were characterised by NTA, with representative NTA profiles showing poly-dispersed profiles of approximately 50–450 nm for EVs isolated from hypoxia-treated and normoxia PC3 cells (Figure 1A). The EVs were confirmed to be positive for CD63 and Flot-1 by Western blotting (Figure 1B and Figure S1) and were further imaged by TEM (Figure 1C). A shift to a smaller modal EV size was observed following hypoxia, compared to normoxia (Figure 1D), and 6 h hypoxia treatment significantly increased total EV release from PC3 cells, compared with normoxic conditions (Figure 1E).

### 3.2. Hypoxic Treatment Increases HIF-1α in EVs and in Recipient Cells Following Hypoxia-EV Transplant

**Figure 2:**
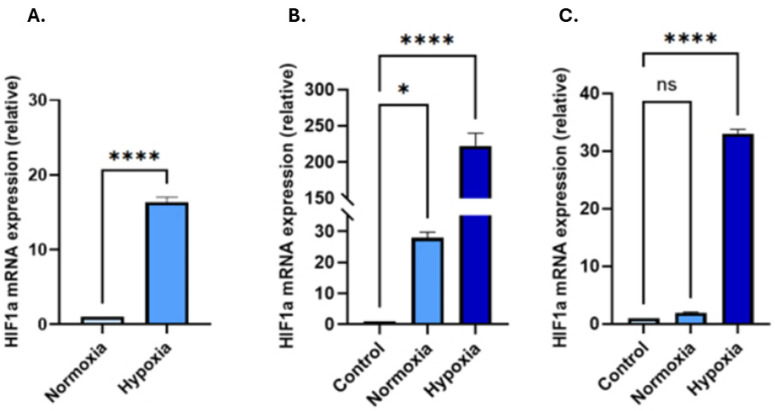
*HIF1-α* expression levels in (**A**) EVs isolated from PC3 cells cultured under normoxic or hypoxic conditions (light blue: PC3 EV normoxia, blue: PC3 EV hypoxia). (**B**) *HIF-1α* mRNA levels in PNT2 and (**C**) LNCaP cells following treatment with PC3-derived EVs compared to untreated cells receiving no EVs. PC3-EVs from hypoxic conditions significantly upregulated HIF-1α expression in both recipient cell lines, compared to untreated cells and cells receiving EVs derived from normoxia-treated PC3 cells. (**B**,**C**: Light blue: untreated cells receiving no EVs; blue: normoxia-EV treated cells; dark blue: hypoxia-EV treated cells). The bar graphs represent the mean of three RNA replicates isolated from control and EV-treated PNT2 and LNCaP cells. Data analysed using two-way ANOVA followed by Šídák’s test. Data normalised according to *RPII* expression and analysed using fold analysis *n* = 3, * *p* ≤ 0.05; **** *p* ≤ 0.0001; ns = not significant; error bars indicate SD.

Changes in HIF-1α mRNA expression in both EVs and in recipient cells exposed to EVs were tested. In the EVs, hypoxia treatment increased the HIF-1α gene expression over 16-fold compared to that found in EVs collected from normoxic PC3 cells (Figure 2A, *n* = 3; *p* < 0.0001). Following 48 h treatment with PC3-derived hypoxic-EVs, a significantly increased HIF-1α expression was observed—by 222-fold in PNT2 cells and by 33-fold in LNCaP cells (*n* = 3; *p* < 0.0001 for both), compared to the untreated cells (Figure 2B,C). Hypoxia treatment, therefore, both elevated *HIF-1α* in PC3 EV cargoes and promoted EV-mediated *HIF-1α* upregulation in recipient cells.

### 3.3. EVs from PC3 Hypoxia-Treated Cells Activate Wnt Signalling and Induce EMT in LNCaP and PNT2 Cells

**Figure 3:**
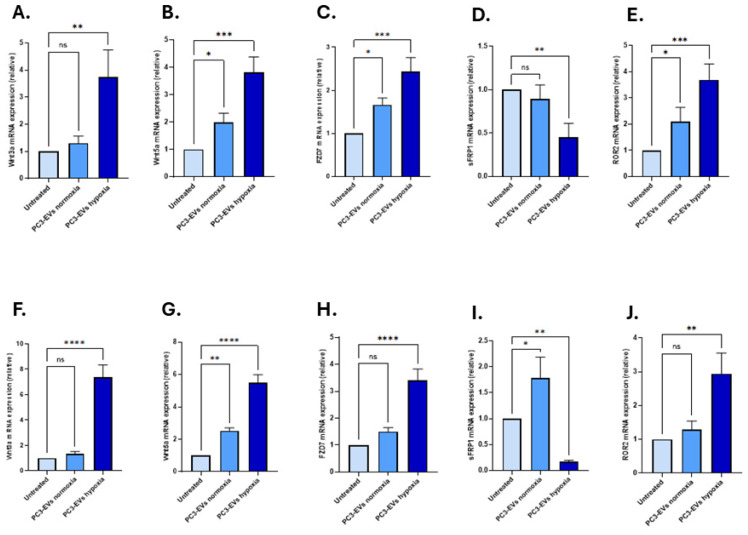
Changes in expression of key Wnt pathway genes following treatment with PC3-derived EVs (**A**–**E**) in PNT2 cells, significant upregulation was detected for *Wnt3a*, *Wnt5a*, *Fzd7*, and *ROR2* gene expression in response to treatment with PC3 hypoxia-EVs. In contrast, *sFRP1* mRNA level was significantly downregulated. (**F**–**J**) in LNCaP cells, PC3 hypoxia-EVs significantly upregulated *Wnt3a*, *Wnt5a*, *Fzd7* and *ROR*2 mRNA levels while *sFRP1* gene expression was significantly downregulated. The bar graphs represent the mean of three RNA replicates isolated from control and EV-treated PNT2 and LNCaP cells. Light blue: untreated cells; blue: normoxia-EV treated; dark blue: hypoxia-EV treated. Data analysed using two-way ANOVA followed by Šídák’s test. Data normalised according to *RPII* expression and analysed using fold analysis, *n* = 3, *p* < 0.05. * *p* ≤ 0.05; ** *p* ≤ 0.01; *** *p* ≤ 0.001; **** *p* ≤ 0.0001; ns = not significant; error bars indicate SD.

To investigate the effects of PC3 hypoxia-induced EVs on Wnt signalling and EMT markers, we measured key target gene expressions following 48 h of EV treatment. PC3-EVs from hypoxic conditions upregulated *Wnt3a*, *Wnt5a*, and *Fzd*7 gene levels in both cell lines compared to treatment with PC3-EVs from normoxic conditions, and untreated controls receiving no EVs (Figure 3A–H). Specifically, in PNT2 cells, *Wnt3a* expression increased to 3.75-fold (*n* = 3, *p* < 0.01), *Wnt5a* by 3.81-fold (*n* = 3, *p* < 0.001) and *Fzd7* by 2.43-fold (*n* = 3, *p* < 0.001), respectively (Figure 3A–C).

In LNCaP cells, *Wnt3a* was significantly upregulated by 7.36-fold (*n* = 3, *p* < 0.0001), Wnt5a by 5.49-fold (*n* = 3, *p* < 0.0001), and Fzd7 by 3.39-fold (*n* = 3, *p* < 0.0001), respectively (Figure 3F–H). In contrast, *sFRP1*, a negative regulator of Wnt signalling, showed varying changes in response to treatment with the PC3-EVs: *sFRP1* was significantly downregulated in PNT2 cells in response to PC3 hypoxia-EVs (0.45-fold; *n* = 3, *p* < 0.01) however application of normoxia-EVs did not result in any significant change (0.89-fold; *n* = 3, *p* > 0.05; Figure 3D). In LNCaP cells, *sFRP1* was upregulated (1.79-fold; *n* = 3, *p* > 0.05) in response to the PC3 normoxia-EVs (*n* = 3, *p* < 0.05); however, significant down-regulation was found in response to treatment with the PC3 hypoxia-EVs (0.17-fold; *n* = 3, *p* < 0.01) (Figure 3I). *ROR2* is a non-canonical Wnt co-receptor, and was significantly upregulated in both cell lines, with a greater increase observed in PNT2 cells (3.70-fold; *n* = 3, *p* < 0.001; Figure 3E,J), compared to LNCaP cells (2.94-fold; *n* = 3, *p* < 0.01; Figure 3J) in response to the PC3 hypoxia-EV treatment.

#### Effects of PC3-Derived EVs from Hypoxia Conditions on EMT Markers

**Figure 4:**
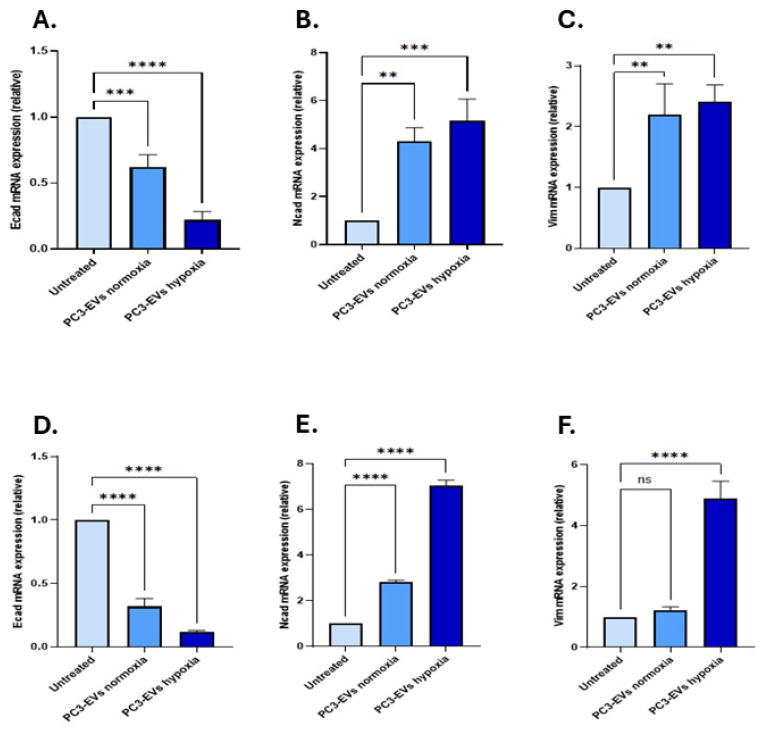
Expression of EMT markers following PC3-derived EV treatments (**A**) *Ecad*, (**B**) *Ncad*, and (**C**) *Vim* mRNA expression levels in PNT2 and (**D**) *Ecad*, (**E**) *Ncad*, and (**F**) *Vim* mRNA expression levels in LNCaP cells. The bar graphs represent the mean of three RNA replicates isolated from PC3-derived EV-treated PNT and LNCaP cells. Light blue: untreated cells; blue: normoxia-EV treated; dark blue: hypoxia-EV treated. Data analysed using two-way ANOVA followed by Šídák’s test. Data normalised according to RPII expression and analysed using fold analysis, *n* = 3, *p* < 0.05. ** *p* ≤ 0.01; *** *p* ≤ 0.001; **** *p* ≤ 0.0001; ns = not significant; error bars indicate SD.

The effects of PC3 EVs isolated from hypoxic conditions on modulating key markers of EMT regulation were evaluated in both PNT2 and LNCaP cells. In PNT2 cells, *Ecad* mRNA levels were significantly downregulated by 0.22-fold (*n* = 3; *p* < 0.0001) following treatment with PC3 hypoxia-EVs, similarly downregulated by 0.62-fold (*n* = 3; *p* < 0.001) following treatment with PC3 normoxia-EVs, both compared with non-treated PNT2 cells (Figure 4A). On the contrary, *Ncad* expression was upregulated by 5.17-fold with hypoxic EV treatment, and by 4.30-fold after normoxia-EVs (*n* = 3; *p* < 0.01; Figure 4B). *Vim* levels were also significantly upregulated following treatment with both hypoxia-EVs (*n* = 3; 2.41-fold, *p* < 0.01) and normoxia-EVs (*n* = 3; 2.19-fold, *p* < 0.01, Figure 4C).

In LNCaP cells, *Ecad* expression was downregulated by 0.12-fold in the hypoxia-EV-treated group and by 0.31-fold in the normoxia-EV-treated group when compared to untreated LNCaP cells (*n* = 3; *p* < 0.0001 for both; Figure 4D). *Ncad* was significantly upregulated by 7.04-fold (*n* = 3; *p* < 0.0001) following PC3 hypoxia-EV application, compared to 2.81-fold in response to normoxia-EV application (*n* = 3; *p* < 0.0001, Figure 4E). In comparison, *Vim* expression was only significantly upregulated following treatment with the PC3 hypoxia-EVs (4.90-fold; *p* < 0.0001), and no significant change was observed following treatment with the PC3-EVs from normoxia conditions compared with untreated cells receiving no EVs (*n* = 3; 1.22-fold; *p* < 0.05, Figure 4F).

### 3.4. PC3-EVs from Hypoxia Conditions Enhance Motility and Invasion of LNCaP PCa Cells

**Figure 5:**
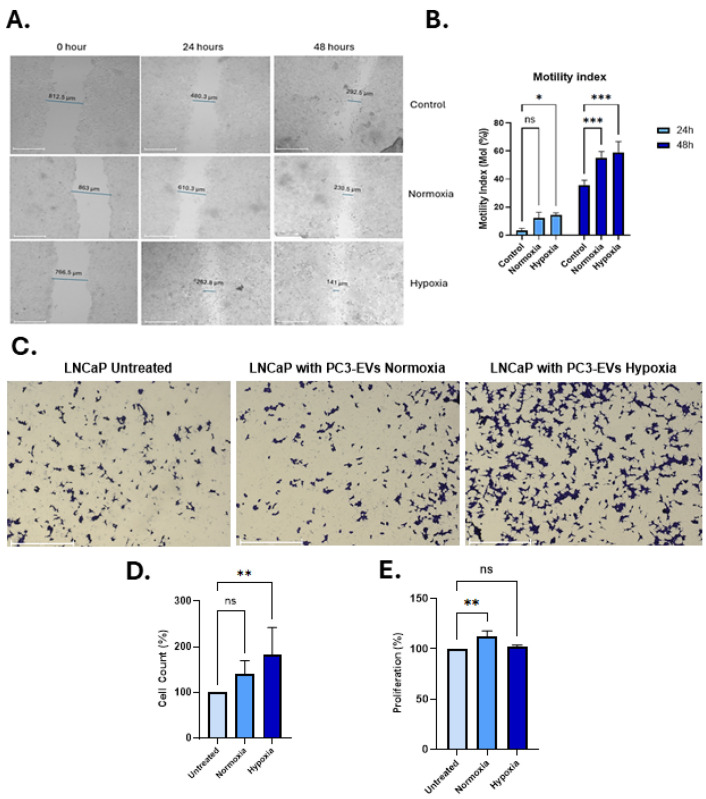
PC3 hypoxia-derived EVs enhance migration and invasion of LNCaP cells. (**A**) Representative wound-healing assay images at 0, 24, and 48 h for untreated cells, and cells treated with PC3 normoxia- or hypoxia-EVs. (**B**) Motility Index quantification shows significantly greater wound closure with hypoxia-EVs at 24 h and 48 h (*n* = 3). (**C**) Representative transwell invasion assay images under the same treatment conditions. (**D**) Quantification confirms significantly increased invasion with hypoxia-EVs versus normoxia-EVs or untreated controls. (**E**) MTT assay revealed no significant proliferation differences, indicating that increased invasion is independent of proliferation. Data analysed using two-way ANOVA followed by Šídák’s test. *n* = 3, * *p* < 0.05, ** *p* < 0.01, *** *p* < 0.001, ns = not significant; error bars indicate SD.

Wound-healing (scratch) assays were performed on LNCaP cells, showing that treatment with PC3-hypoxia significantly accelerated wound closure over a 48 h period, compared to cells treated with normoxic PC3-EVs, or untreated controls (receiving no EVs) (Figure 5A,B). This indicates enhanced cancer cell migratory capacity mediated by the hypoxia-conditioned PC3-EVs. This was further supported by results from the transwell invasion assays, showing a significant increase in the number of invading LNCaP cells following treatment with the PC3 hypoxia-EVs (Figure 5C,D). Importantly, the PC3-hypoxia-EVs did not significantly affect proliferation rates, compared with control treatments (Figure 5E), confirming that the observed increase in invasion was not due to enhanced cell proliferation. On the other hand, PC3 normoxia EVs increased proliferation slightly in LNCaP cells; however, there was no effect on invasion (Figure 5C,E).

## 4. Discussion

PCa remains a major clinical challenge due to its molecular heterogeneity, unpredictable progression, and high metastatic potential [59]. Among the many factors influencing PCa progression, hypoxia has emerged as a critical determinant of the tumour microenvironment in PCa, similar to other solid malignancies [30,44]. Hypoxia induces cellular adaptation largely through hypoxia-inducible factors, particularly HIF-1α, which drive the transcription of genes involved in angiogenesis, metabolic reprogramming, and EMT, thereby enhancing cancer cell invasiveness and resistance to therapy [60]. Notably, HIF-1α activation promotes EMT by inducing EMT-driving transcription factors and cadherin switching, thereby enhancing cell motility and invasiveness [61,62]. Furthermore, HIF-1α signalling crosstalk with Wnt/β-catenin pathways has been reported, with hypoxia-induced HIF-1α shown to cooperate with β-catenin, mutually amplifying Wnt/β-catenin transcriptional activity [63,64]. Chronic hypoxia can drive PCa cells towards an androgen-independent state, accompanied by widespread transcriptional and metabolic reprogramming characteristic of CRPC [65]. Both in vivo and clinical studies have linked intratumoural hypoxia to treatment failure, with HIF-1α overexpression in PCa correlating not only with resistance to androgen-deprivation therapy but also with reduced radiosensitivity. Hypoxic tumour regions are inherently less responsive to radiotherapy due to limited oxygen availability for radiation-induced DNA damage, contributing to poorer clinical outcomes [65,66].

EVs are crucial mediators of tumour–stromal communication, capable of transferring oncogenic proteins, RNAs, and lipids to reprogram recipient cells [25,67,68,69]. Hypoxia enhances both EV secretion and alters cargo composition, often conferring more aggressive phenotypes [70]. In this study, PC3 hypoxia-EVs significantly elevated HIF-1α expression in PNT2 and LNCaP cells after 48 h, further implicating hypoxia-driven EV signalling in pro-oncogenic pathway activation. We also provide evidence that EVs secreted by hypoxia-conditioned metastatic PC3 cells may promote Wnt signalling and EMT gene expression in non-tumorigenic, PNT2 and malignant LNCaP prostate epithelial cells. PC3-derived hypoxic EVs upregulated *Wnt3a*, *Wnt5a*, and *Fzd7* in both LNCaP and PNT2 recipient cells, alongside increased expression of mesenchymal markers *Ncad* and *Vim* and decreased *Ecad* expression. Our data are consistent with hypoxia-induced migratory and invasive phenotypes previously reported in PCa [43]. Moreover, previous studies have identified that hypoxia-associated miRNAs, such as miR-21, miR-145 and miR-210, drive similar phenotypic changes, which repress Ecad and promote EMT [25,61,67]. Our findings also align with reports from other tumour types, including breast and colorectal cancer, where hypoxia-derived EVs have been shown to activate the Wnt pathway and remodel the TME [71,72]. Hypoxia may also enhance EV biogenesis via altered lipid metabolism and stimulate matrix remodelling through MMPs [25,73]. In addition to reprogramming recipient epithelial cells, hypoxia-induced EVs were also reported to activate stromal components, including cancer-associated fibroblasts (CAFs), thereby amplifying their pro-tumorigenic roles [74]. Stromal modifications can contribute to a permissive tumour microenvironment characterised by immune evasion, angiogenesis, and ECM remodelling [75]. Notably, EVs from hypoxic PCa cells have been shown to deliver VEGF and Rab5-activating guanine exchange factors such as ALS2, which destabilise the β-catenin destruction complex in endothelial cells, stabilising nuclear β-catenin and enhancing Wnt target gene activation [25,76]. Importantly, in this current study, we noted a significant upregulation of the canonical ligand *Wnt3a*, the non-canonical ligand *Wnt5a*, and the receptor *Fzd7* in both PNT2 and LNCaP cells upon treatment with hypoxic PC3-EVs. This broad activation of the Wnt machinery underscores its importance as a downstream effector of EV-mediated signalling. However, analysis of the specific components regulated, particularly the Wnt5a/ROR2 axis and the inhibitor sFRP1 reported here, reveals a profound and context-dependent complexity that offers insights into the mechanisms of PCa progression.

In our present study, hypoxic-EVs upregulated ROR2, a non-canonical Wnt co-receptor, particularly in the PNT2 cells. sFRP1, a Wnt antagonist, was variably regulated, suggesting that EVs may override inhibitory Wnt mechanisms to trigger oncogenesis [14,19,22]. The stronger transcriptional response in LNCaP cells to hypoxia-EVs may reflect epigenetic and transcriptional priming in malignant cells, including altered chromatin accessibility and TF activity [77,78,79,80]. Upregulation of Fzd7 and ROR2 by hypoxia-EVs may be observed here suggesting dual activation of canonical and non-canonical Wnt signalling. Fzd7 is linked to therapy resistance and stemness, while ROR2 promotes migration via cytoskeletal reorganisation. Notably, ROR2 may exhibit context-dependent functions in PCa, acting as either a tumour suppressor or a mediator of therapeutic resistance [13,22]. Likewise, sFRP1 can act as a tumour suppressor in epithelial cells but supports tumour growth via stromal expression [81]. The identified upregulation here of both Wnt5a and its co-receptor ROR2 is particularly intriguing due to their dual and often contradictory roles in cancer. In many contexts, including the primary tumour setting investigated in this study, the Wnt5a/ROR2 axis is a potent driver of EMT, cytoskeletal rearrangement, and invasion [82]. The enhanced migration and invasion of LNCaP cells following hypoxic EV treatment observed here correlate directly with the upregulation of this axis, supporting its pro-metastatic function. However, a separate and compelling body of evidence has shown that in the unique microenvironment of the bone, Wnt5a secreted by osteoblasts acts via ROR2 to suppress canonical Wnt/β-catenin signalling, thereby inducing a state of dormancy in disseminated tumour cells [82]. This duality in function is not a contradiction but rather a key insight into tumour cell adaptability. It can be postulated that hypoxic EVs equip cancer cells with the Wnt5a/ROR2 signalling machinery necessary for dynamic adaptation to different microenvironments. The same pathway that fuels invasion and escape from the primary tumour can possibly be co-opted by signals within the bone niche to switch the cell to a dormant, survival-oriented state. This model provides a plausible explanation for the clinical phenomenon of metastatic latency, where cancer cells can survive for years in a quiescent state before reawakening to form overt metastases, and will need further in-depth investigation.

Prior reports show that EVs can convert stromal fibroblasts into CAFs via activation of chemokine and cell cycle pathways, such as CCL2 and CXCL8 [79]. These processes may underpin LNCaP susceptibility to EV-driven phenotypic shifts. EV cargoes, including ncRNAs and histone modifiers, can remodel recipient cell epigenomes, repress tumour suppressors, and activate Wnt/EMT-linked genes [83,84]. LNCaP cells, characterised by AR and ETS factor dysregulation, may be particularly sensitive to such mechanisms [85,86], whereas PNT2 cells likely remain intact regulatory circuits [87].

The induction of HIF-1α, Ncad, and Vim, which was observed in response to PC3 hypoxia-EV treatment in this study, may be correlated with reported adaptive responses facilitating metastasis, EMT remodelling, and resistance to conventional therapy [88]. In PTEN-deficient mouse models of PCa, HIF-1α activation has been detected early during prostatic intraepithelial neoplasia, coinciding with the onset of hyperplasia when cell proliferation begins to exceed oxygen supply [89]. This supports the concept that hypoxia-driven signalling is initiated at pre-malignant stages and can influence disease progression. Through EVs and other signalling molecules, hypoxic cell clones may remodel the surrounding microenvironment, creating niches that reinforce hypoxia, promote clonal diversification, and contribute to tumour heterogeneity. Our current data align with previous studies showing the potential of tumour-derived EVs in promoting aggressive cancer phenotypes [90].

Several study limitations should be acknowledged, including the use of 2D monolayer cultures, which cannot fully reproduce the structural complexity, extracellular matrix composition, or cell–cell interactions of the prostate TME. It will need to be assessed whether EVs derived from other PCa cell lines, including molecular subtypes, and at different disease stages, show similar results to the PC3-EVs. Future work using 3D prostate organoid models, derived from both malignant and benign tissues, and in vivo xenograft and genetically engineered mouse models, will need to be used to evaluate whether hypoxia-derived EVs influence tumour progression within an intact microenvironment. In parallel, further comprehensive profiling of EV cargoes under hypoxic conditions, in addition to HIF1α identified here, will need to be carried out.

Clinically, the upregulation of Fzd7 and ROR2 by hypoxia-EVs, identified here, highlights potential therapeutic avenues. Fzd7 inhibitors, monoclonal antibodies against ROR2, and small-molecule inhibitors of EV biogenesis or uptake are under active investigation in several cancers [91,92,93,94,95] and may offer combinatory strategies with androgen deprivation and/or chemotherapy to mitigate EV-mediated pro-metastatic signalling.

## 5. Conclusions

This study identifies the roles of EVs released from hypoxic PCa cells in activating Wnt and EMT pathways in both malignant and non-malignant prostate cells, potentially contributing to tumour heterogeneity and disease progression. The reported mRNA-level changes, characterised by reduced Ecad and increased Ncad and Vim expression, are consistent with a shift towards a more motile, and hence aggressive, PCa phenotype. Our findings highlight hypoxia-induced EV signalling as a promising therapeutic target and for biomarker discovery in PCa.

## Figures and Tables

**Table 1:** Sequences of the primers utilised in qRT-PCR.

Primer	Forward Sequence (5’-3’)	Reverse Sequence (5’-3’)
HIF1α	TTCACAAATCAGCACCAAGC	TGCAACATGGAAGGTATTGC
Wnt3a	GTTGGGCCACAGTATTCCTC	ATCCCACCAAACTCGATGTC
Wnt5a	TCTCAGCCCAAGCAACAAGG	GCCAGCATCACATCACAACAC
Fzd7	GTCACGGATGCTGTTATTAAGG	CACATCGCCGTTATCATCATC
sFRP1	CAATGCCACCGAAGCCTCCAAG	CAAACTCGCTGGCACAGAGATG
ROR2	TTTGTGCGGCTGGGTCCAA	GTAAGGCTGGCAGAACCCAT
Ecad	AAGAAGCTGGCTGACATGTACGGA	CCACCAGCAACGTGATTTCTGCAT
Ncad	CCTCCAGAGTTTACTGCCATGAC	GTAGGATCTCCGCCACTGATTC
Vim	TACAGGAAGCTGCTGGAAGG	ACCAGAGGGAGTGAATCCAG
RPII	GCACCACGTCCAATGACAT	GTGCGGCTGCTTCCATAA

qRT-PCR was performed using SYBR Green Master Mix (QuantiTect SYBR Green PCR Master Mix, Qiagen, Manchester, UK). The thermocycling conditions were set as follows: 95 °C for 2 min, followed by 95 °C for 10 s and 56 °C for 60 s, as previously described [54,55]. The comparative 2^−ΔΔCT^ method was used to determine the relative mRNA expression levels, with *RPII* serving as the reference gene [53,56].

## Data Availability

The original contributions presented in the study are included in the article/Supplementary Materials, further inquiries can be directed to the corresponding authors.

## Supplementary Materials

The following supporting information can be downloaded at: https://www.mdpi.com/article/10.3390/biology14091135/s1, Figure S1: Original protein image.

## Abbreviations

The following abbreviations are used in this manuscript:

ADT	Androgen Deprivation Therapy
AR	Androgen Receptor
CAFs	Cancer-Associated Fibroblasts
CSC	Cancer Stem Cell
CRPC	Castration-Resistant Prostate Cancer
CXCL8	C-X-C Motif Chemokine Ligand 8 (also known as Interleukin-8)
DAPI	4′,6-Diamidino-2-Phenylindole (a fluorescent DNA stain)
DMEM	Dulbecco’s Modified Eagle Medium
ECM	Extracellular Matrix
EMT	Epithelial-to-Mesenchymal Transition
EVs	Extracellular Vesicles
FBS	Fetal Bovine Serum
Fzd7	Frizzled Class Receptor 7
Glut1	Glucose Transporter 1
HIF-1α	Hypoxia-Inducible Factor 1-alpha
lncRNA	Long Non-Coding RNA
LNCaP	Lymph Node Carcinoma of the Prostate (a prostate cancer cell line)
miRNA	MicroRNA
MMP2	Matrix Metalloproteinase 2
MMP9	Matrix Metalloproteinase 9
MTT	3-(4,5-Dimethylthiazol-2-yl)-2,5-Diphenyltetrazolium Bromide (a cell viability assay)
N-cad	N-cadherin (Neural cadherin)
PBS	Phosphate-Buffered Saline
PC3	Prostate Cancer Cell Line 3
PCa	Prostate Cancer
PHD	Prolyl Hydroxylase Domain (enzymes)
PI3K	Phosphatidylinositol 3-Kinase
PNT2	Prostate Normal Tissue 2 (non-tumorigenic prostate epithelial cell line)
qPCR	Quantitative Polymerase Chain Reaction
ROR2	Receptor Tyrosine Kinase-Like Orphan Receptor 2
sFRP1	Secreted Frizzled-Related Protein 1
VEGF	Vascular Endothelial Growth Factor
Vim	Vimentin
VHL	von Hippel–Lindau Protein
Wnt	Wingless/Integrated (signalling pathway)
