# Supplementary figures and images for: Hypoxia-Driven Extracellular Vesicles Promote Pro-Metastatic Signalling in LNCaP Cells via Wnt and EMT Pathways
Source: Biology (Basel). 2025 Aug 27;14(9):1135. doi: 10.3390/biology14091135 (PMC12467610; doi:10.3390/biology14091135)

## Slide 1
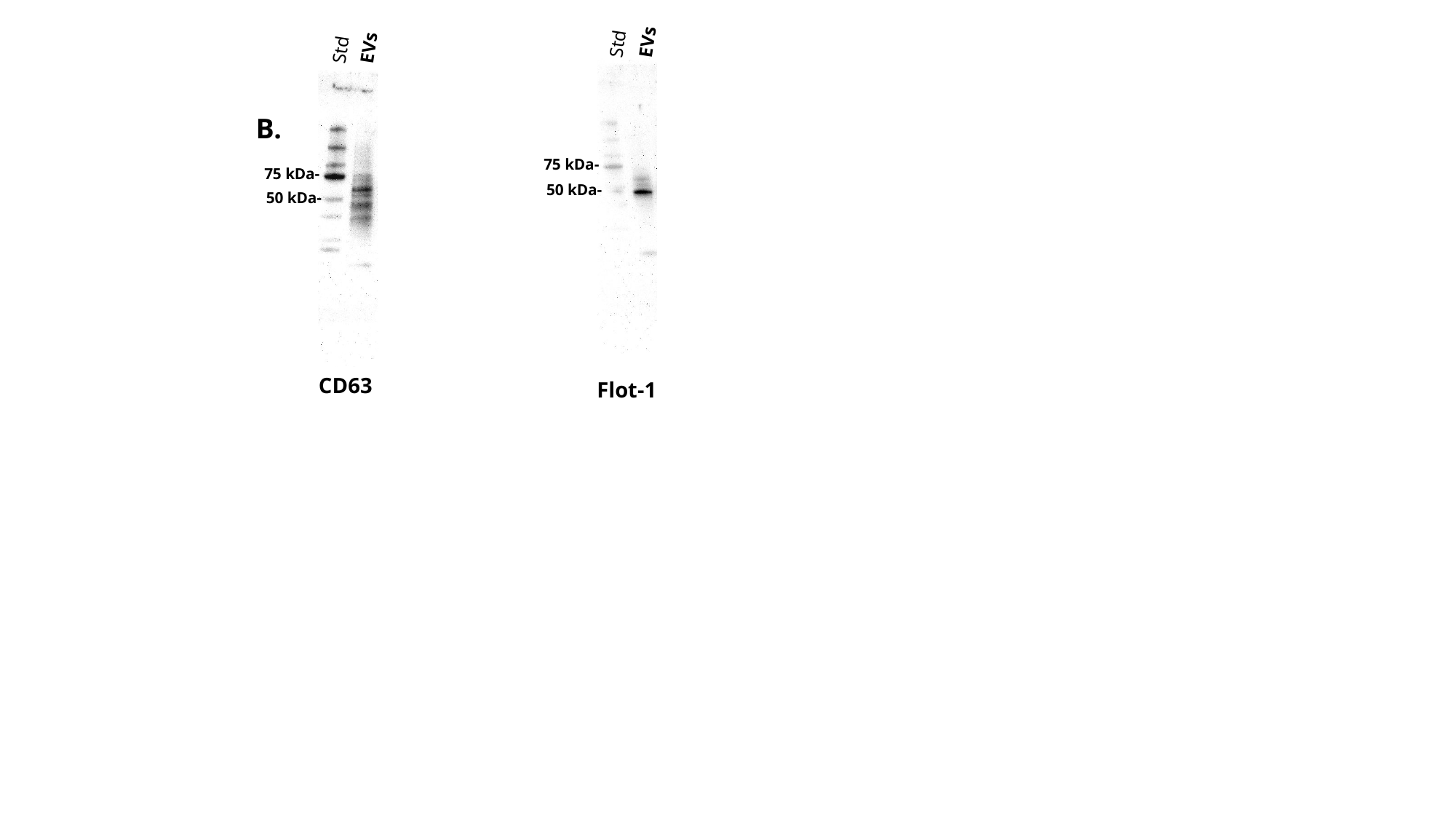

EVs
Std
EVs
Std
B.
75 kDa-
75 kDa-
50 kDa-
50 kDa-
CD63
Flot-1

Supplement: Supplementary file 1 [file biology-14-01135-s001.zip › biology-3816449 Supplementary/Hypoxia EVs WB full blots SL 2.pptx]
